# Effects of implementing rotational thromboelastometry in cardiac surgery: A retrospective cohort study

**DOI:** 10.1177/1750458920950662

**Published:** 2021-01-10

**Authors:** Minna Kallioinen, Mika Valtonen, Marko Peltoniemi, Ville-Veikko Hynninen, Tuukka Saarikoski, Oki Söderholm, Tommi Kauko, Jenni Aittokallio

**Affiliations:** 1 Division of Perioperative Services, Intensive Care Medicine and Pain Management, Turku University Hospital and University of Turku, Turku, Finland; 2 Department of Anesthesiology and Intensive Care, University of Turku, Turku, Finland; 3 Auria Clinical Informatics, Turku University Hospital, Turku, Finland

**Keywords:** Cardiothoracic surgery / Clinical investigations / Patient safety/outcomes / Management of bleeding / Operating theatre

## Abstract

Since 2013, rotational thromboelastometry has been available in our hospital to assess coagulopathy. The aim of the study was to retrospectively evaluate the effect of thromboelastometry testing in cardiac surgery patients. Altogether 177 patients from 2012 and 177 patients from 2014 were included. In 2014, the thromboelastometry testing was performed on 56 patients. The mean blood drainage volume decreased and the number of patients receiving platelets decreased between 2012 and 2014. In addition, the use of fresh frozen plasma units decreased, and the use of prothrombin complex concentrate increased in 2014. When studied separately, the patients with a thromboelastometry testing received platelets, fresh frozen plasma, fibrinogen and prothrombin complex concentrate more often, but smaller amounts of red blood cells. In conclusion, after implementing the thromboelastometry testing to the practice, the blood products were given more cautiously overall. The use of thromboelastometry testing was associated with increased possibility to receive coagulation product transfusions. However, it appears that thromboelastometry testing was mostly used to assist in management of major bleeding.

**Provenance and Peer review:** Unsolicited contribution; Peer reviewed; Accepted for publication 26 July 2020.

## Introduction

Severe bleeding and coagulopathy are critical but common complications in cardiac surgery. Transfusion of allogeneic blood products is associated with multiple adverse events, including increased risk of postoperative renal failure, pulmonary complications, infection, neurological complications and mortality at 30 days (Koch et al 2006, Murphy et al 2007, Likosky et al 2015) and longer, even five years after surgery (Engoren et al 2002). It is therefore essential to discover new measures to decrease transfusion rates, as well as to manage bleeding and coagulation. In recent years, viscoelastic point-of-care whole blood tests have addressed this need and are increasingly used in the perioperative period to guide transfusion management (Whiting & DiNardo 2014), however there are also conflicting aspects of their usefulness (Hunt et al 2015).

Viscoelastic tests, like thromboelastography and rotational thromboelastometry (ROTEM), are tools for rapidly evaluating clot formation. Both methods produce results by showing changes in the viscoelastic strength of a small blood sample, allowing visual assessment of the formation and lysis of the clot at the point of care. Many centres have developed their own protocols and algorithms using these methods to assess coagulation and to guide the treatment (Ichikawa et al 2018, Mehaffey et al 2017). In the operating theatre, the faster turn-around time of ROTEM favours it over traditional laboratory testing (Olde Engberink et al 2014). A Cochrane review published in 2016 concluded that the existing trial evidence was insufficient to demonstrate that viscoelastic testing improved clinical outcomes (Wikkelsø et al 2016). Since then, Karkouti et al (2016) published a large trial in 2016 involving 7402 cardiac surgery patients in 12 Canadian hospitals and another two meta-analyses on the subject have been conducted afterwards (Serraino & Murphy 2017, Wikkelsø et al 2017). To summarise the evidence, the use of viscoelastic test based algorithms undeniably reduced the number of patients requiring transfusion but had no effect on mortality, stroke, prolonged intubation, emergency re-operation for bleeding or in intensive care unit (ICU) and the hospital length of stay (Serraino and Murphy 2017). However, a significant reduction in the frequency of severe acute kidney injury was seen (Serraino & Murphy 2017, Wikkelsø et al 2017) and a trend towards reduced mortality, but the quality of the evidence is low (Wikkelsø et al 2017).

Our cardiac surgery unit is a low volume unit with approximately 450 cardiac surgery patients per year. ROTEM was introduced in our hospital in 2013 and very quickly became a widely used method of evaluating coagulopathy intraoperatively in our cardiac surgery patients who had severe bleeding. Since then it has been in our interest to retrospectively evaluate, if the introduction and implementation of ROTEM into our practice has changed the usage of blood products and coagulation factors in our operating unit and, in addition, if it has had an effect on the re-operation rate, postoperative blood loss via chest drains (drainage volume) or the length of stay at the ICU. The aim of this study is to retrospectively compare the care of cardiac patients from two different time periods: before and after the introduction of ROTEM. We wanted to evaluate the impact of the method in our practice and to determine its usefulness for the cardiac surgery patients.

## Methods

### Data sources and study population

The data of 408 surgical cardiac patients were collected. The data were gathered retrospectively from two different six-month periods: from January 2012 to June 2012 and from January 2014 to June 2014. The individual patient data were collected from the hospital’s patient documents with the permission of the Turku Clinical Research Centre Ethical Committee (project number T303/2017). The data from the hospital software were combined and all patient data including name, identification number and day of surgery were deidentified prior to statistical analyses. The following preoperative data were collected: age, sex, haemoglobin (Hb), platelet count, international normalised ratio (INR), left ventricular ejection fraction (EF) and the usage of anticoagulants and antiplatelet drugs. Anticoagulants and antiplatelet drugs considered were warfarin, aspirin, clopidogrel, low molecular weight heparin (LMWH) and direct oral anticoagulant (DOAC). All the operations were done on cardiopulmonary bypass. The time on cardiopulmonary bypass (perfusion time) and the ischaemic time of the heart (aortic closure time) were collected. After operation the patients were transferred to the ICU. The ICU length of stay and postoperative drainage volume was gathered up from the ICU patient documents. In addition, the usage of blood products including red blood cells, fibrinogen, fresh frozen plasma (FFP), platelets and prothrombin complex concentrate (PCC) during and after operation was analysed.

**Figure 1:**
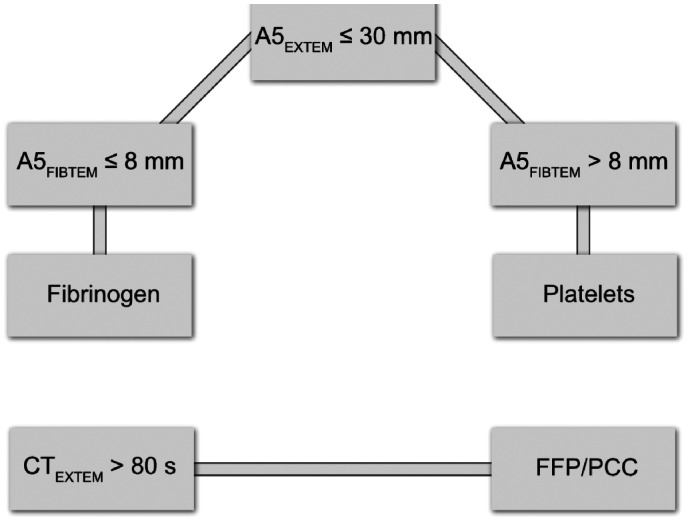
The ROTEM-guided transfusion protocol. A5: describe the clot firmness (amplitude) after 5 minutes; CT: clotting time; EXTEM: a screening test for the extrinsic haemostasis system; FIBTEM: EXTEM based assay for the fibrin part of the clot. Two analyses were always done: EXTEM and FIBTEM. Fresh frozen plasma or prothrombin complex concentrate were given if CT of EXTEM curve was over 80s. EXTEM was checked before FIBTEM. A5 is the amplitude of the curve after 5 minutes. If the amplitude of the EXTEM (A5EXTEM) was under 30mm, the A5FIBTEM was checked and either fibrinogen or platelets were given, depending on the A5FIBTEM results (A5FIBTEM ≤8 → fibrinogen or A5FIBTEM >8 → platelets)

The usage of ROTEM testing was evident from the patient’s documents. In our surgical department, the transfusions were determined by the anaesthetists according to their evaluation of the blood loss with the help of bedside Hb measurements, laboratory assessments and patient monitoring. If ROTEM was used, we had a specific protocol to determine the need and type of the coagulation product (Figure 1). Red blood cells were given according to the Hb measurements.

The patients from 2012 and 2014 were paired and matched according to age, sex and operation type. One hundred and seventy-seven matched pairs were found, after 54 patients from the original total of 408 were excluded due to massive drainage, missing values or a lack of a similar pair. In addition, from the year 2014 the 56 patients with proper records of ROTEM testing (the ROTEM group) were matched according to age, sex and operation type with 56 patients from year 2012 (the control group).

### Statistical methods

Due to extreme skewness in distribution (massive drainage), the 2.5% of highest drainages in ml were excluded. Continuous measurement of INR was split into two classes, below or equal to 1.2 unit and greater than 1.2 units. Demographics were tabulated from the whole patient population. Statistically significant differences were tested using *t*-test, Mann–Whitney *U*-test, *χ*^2^-test and Fisher’s exact test for continuous and normally distributed variables, continuous and not normally distributed variables, count variables meeting expected cell count assumption and count variables which do not meet assumptions for *χ*^2^-test, respectively. Individual patients were paired using propensity score matching with the nearest neighbour method. Patients were matched using their demographic qualities of age, sex and operation type. General linear models and zero-inflated Poisson regression models were fitted to data for each continuous and count response variable individually using propensity scores as weights, respectively. In addition, if data were over dispersed, zero-inflated negative-binomial regression model was fitted instead of zero-inflated Poisson regression model. Usage of zero-inflated Poisson regression models over ordinary Poisson regression models was justified with Vuong’s non-nested hypothesis test. Models included year and anticoagulation medications as independent variables. All *p*-values less than 0.05 were considered as statistically significant. All analyses were performed using R version 3.6.1 with MatchIt version 3.0.2, cem version 1.1.19 and pscl version 1.5.2 libraries.

## Results

### Patient characteristics in 2012 and 2014

After matching, overall 354 cardiac surgery patients were included in the analyses; 177 from year 2012 and 177 from year 2014. The mean age was 66.5 years in 2012 cohort and 65.8 years in 2014 cohort, respectively (*p* = 0.602). There were fewer women in either cohort, 32.5% and 28.3%, respectively (*p* = 0.487). The rate of early postoperative re-sternotomies was similar: 5.5% in the 2012 cohort and 7.9% in the 2014 cohort (*p* = 0.457). There was no difference in preoperative Hb, platelet count, INR or EF between the two years. The mean perfusion times were similar: 102min in the 2012 cohort and 101min in the 2014 cohort (*p* = 0.808). Also, the proportion of patients with ongoing warfarin, aspirin and DOAC therapy was similar (no medication discontinuation before surgery). However, in the 2014 cohort, the proportion of patients with ongoing clopidogrel and LMWH was higher than in the 2012 cohort (11% vs 24%; *p* = 0.033 and 15% vs 32%; *p* = 0.003, respectively). The mean ICU length of stay was 43h in the 2012 cohort and 39h in the 2014 cohort (*p* = 0.5596). The median ICU stays were 23h in the 2012 cohort and 24h in the 2014 cohort, respectively. Only 14.1% of all the patients stayed in the ICU for longer than three days (Table 1).

**Table 1:** Demographic data of the 2012 cohort and 2014 cohort

	2012 cohort	2014 cohort	*p*-Value
*N*	177	177	1.0000^χ^
Age (mean, SD)	66.5 (10.7)	65.8 (11.5)	0.6024^t^
Women (*n*, %)	57 (32.2)	50 (28.2)	0.4874^χ^
Hb (mean, SD)	135.7 (18.4)	135.2 (19.6)	0.8167^t^
Platelets (mean, SD)	211.2 (51.5)	219.6 (58.3)	0.1527^t^
INR (mean, SD)	1.2 (0.4)	1.2 (0.4)	0.8345^t^
EF (mean, SD)	54.2 (11.3)	55.9 (11.9)	0.0714^U^
Perfusion time (min) (mean, SD)	102.0 (47.7)	100.8 (48.5)	0.8080^t^
Warfarin (*n*, %)	26 (14.7)	21 (11.9)	0.5310^χ^
Aspirin (*n*, %)	45 (25.4)	62 (35.0)	0.0641^χ^
Clopidogrel (*n*, %)	11 (6.2)	24 (13.6)	**0.0326**^χ,^*
LMWH (*n*, %)	15 (8.5)	32 (18.1)	**0.0122**^χ,^*
DOAC (*n*, %)	1 (0.6)	0 (0.0)	1.0000^F^
Early re-sternotomy (*n*, %)	9 (5.1)	14 (7.9)	0.3883^χ^
ICU stay (h) (median, SD)	23.0 (101.1)	24.0 (49.8)	0.1017

DOAC: direct oral anticoagulant; EF: ejection fraction; Hb: haemoglobin; ICU: intensive care unit; INR: international normalised ratio; LMWH: low molecular weight heparin.

t = *t*-test, U = Mann–Whitney *U*-test, χ = *χ*^2^-test and F = Fisher’s exact test.

* Statistically significant.

### Patient characteristics of the ROTEM patients

Altogether 56 patients in the 2014 cohort had records of ROTEM usage in the patient documents. According to the patient documents, ROTEM was mostly used to evaluate coagulopathy in the case of severe bleeding. The ROTEM patients were matched in terms of age, sex and operation type with similar pairs from 2012. The mean age in the ROTEM group was 66.4 years and in the control group 67.4 years (*p* = 0.613). The groups did not differ in terms of preoperative Hb, platelet count, EF, perfusion time or operation type. The amount of early postoperative re-sternotomies was similar: 7.1% in the ROTEM group and 8.9% in the control group (*p* = 0.742). The mean ICU time in the ROTEM group was 37.8 hours and 64.9 hours in the control group, and the median ICU time was 24 hours and 24.5 hours, respectively (*p* = 0.016). Moreover, in the ROTEM group, the number of clopidogrel users was markedly higher (*p* = 0.016) (Table 2).

**Table 2:** Demographic data of the ROTEM group and the control group

	ROTEM group	Control group	*p*-Value
*N*	56	56	1.0000^χ^
Age (mean, SD)	66.4 (10.2)	67.4 (11.2)	0.6129^t^
Women (*n*, %)	15 (26.8)	12 (21.4)	0.6586^χ^
Hb (mean, SD)	134.6 (19.3)	134.2 (20.5)	0.9097^t^
Platelets (mean, SD)	222.9 (66.6)	218.9 (54.7)	0.7245^t^
INR (mean, SD)	1.3 (0.6)	1.2 (0.4)	0.2732^t^
EF (mean, SD)	54.8 (12.8)	53.0 (11.0)	0.4246^t^
Perfusion time (min) (mean, SD)	120.1 (49.9)	119.8 (44.8)	0.9715^t^
Warfarin (*n*, %)	12 (21.4)	8 (14.3)	0.4592^χ^
Aspirin (*n*, %)	22 (39.3)	27 (48.2)	0.4461^χ^
Clopidogrel (*n*, %)	16 (28.6)	5 (8.9)	**0.0155**^χ,^*
LMWH (*n*, %)	16 (28.6)	13 (23.2)	0.6662^χ^
DOAC (*n*, %)	1 (1.8)	1 (1.8)	1.0000^F^
Early re-sternotomy (*n*, %)	4 (7.1)	5 (8.9)	0.7422^F^
ICU stay (h) (median, SD)	24.0 (40.2)	24.5 (116.6)	**0.0556**^t,^*
CABG (*n*, %)	25 (44.6)	29 (51.8)	0.5705^χ^
CABG + valve replacement (*n*, %)	6 (10.7)	4 (7.1)	0.7404^χ^
Aortic valve replacement (*n*, %)	14 (25.0)	10 (17.9)	0.4897^χ^
Mitral valve replacement (*n*, %)	5 (8.9)	6 (10.7)	1.0000^χ^
Ascended aortic replacement with or without aortic valve replacement (*n*, %)	9 (16.1)	8 (14.3)	1.0000^χ^
Other (*n*, %)	2 (3.6)	1 (1.8)	1.0000^F^

CABG: coronary artery bypass grafting; DOAC: direct oral anticoagulant; EF: ejection fraction; Hb: haemoglobin; ICU: intensive care unit; INR: international normalised ratio; LMWH: low molecular weight heparin.

t = *t*-test, U = Mann–Whitney *U*-test, χ = *χ*^2^-test and F = Fisher’s exact test.

* Statistically significant.

### Postoperative bleeding

**Figure 2:**
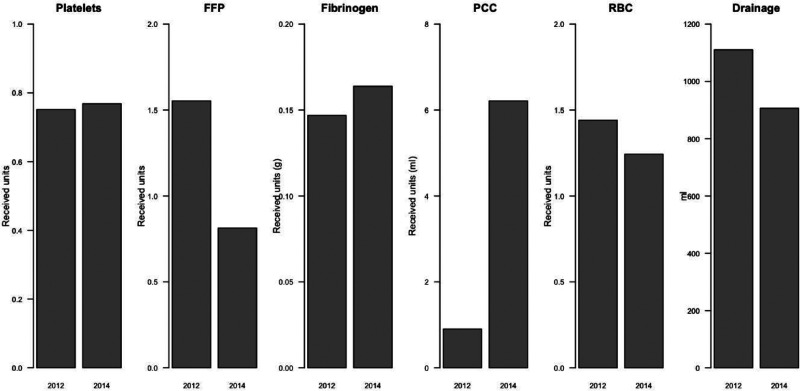
The usage of blood products and the average postoperative bleeding (drainage) in 2012 and 2014. FFP: fresh frozen plasma; PCC: prothrombin complex concentrate; RBC: red blood cells

The postoperative blood drainage volume was found to have significantly decreased in the 2014 cohort compared to the 2012 cohort (826ml, SD = 1655 vs 1064ml, SD = 2059; *p* = 0.006, Figure 2). Between the ROTEM group and the control group, there was no statistical difference in the overall postoperative drainage (1083ml vs 1333ml, *p* = 0.392, Table 3). When examining the operation types separately, in the combination operation of CABG and valve replacement the difference was 2678ml less bleeding in the ROTEM group than in the control group (*p* = 0.005, Table 3). The preoperative use of anticoagulants or antiplatelet drugs did not affect the amount of postoperative drainage in any of the patients.

**Table 3:** The postoperative blood drainage after operation in the ROTEM group and in the control group

	Drainage		
Procedure	Control group	Change to ROTEM group	*p*-Value
All	1333 (207)	–250 (291)	0.3920
CABG	1197 (284)	240 (417)	0.5641
CABG + valve replacement	3485 (742)	–2678 (958)	**0.0052** *
Aortic valve replacement	977 (513)	–142 (658)	0.8290
Mitral valve replacement	839 (629)	–65 (933)	0.9443
Aortic replacement with or withoutaortic valve replacement	1422 (546)	–608 (751)	0.4182
Other	755 (1544)	–389 (1891)	0.8370

CABG; coronary artery bypass grafting.

Linear models were used. Data are presented as ml, mean (SD).

* Statistically significant.

### Blood product utilisation

Red blood cell usage did not differ between the 2012 and the 2014 cohorts: a similar number of patients received red blood cells (71 vs 68 patients; *p* = 0.617) and in similar amounts (3.9 vs 3.2 units/patient; *p* = 0.065). Likewise, with fibrinogen there was no change between the 2012 and the 2014 cohorts (12 vs 12 patients; *p* = 0.854 and 2.6 vs 2.6 units/patient; *p* = 0.910). The total amount of platelet units used did not change between the 2012 and the 2014 cohorts (*p* = 0.074, Figure 2), but the number of patients who received platelets decreased (57 vs 50 patients; *p* = 0.003). The FFP units were given less frequently in 2014 (61 vs 34 patients; *p* = 0.001). In 2012, 34.5% of patients received FPP, whereas in 2014 the percentage was 18.6%. The usage of PCC increased significantly from the 2012 cohort to the 2014 cohort; it was given more often (7 vs 26 patients; *p* < 0.001) and in greater amounts (2.9 vs 4.1 units/patient; *p* < 0.001) in 2014. In 2012, only 4% of patients received PCC whereas in 2014 the share was 14.7% (Table 4 and Figure 2).

**Table 4:** Blood product usage in the 2012 cohort and in the 2014 cohort

Count part Logit part	Platelets (units) ^a^		FFP (units) ^b^		Fibrinogen (units) ^a^		PCC (units) ^a^		RBC (units) ^b^	
	OR (CI)	*p*-Value	OR (CI)	*p*-Value	OR (CI)	*p*-Value	OR (CI)	*p*-Value	OR (CI)	*p*-Value
Year 2012 (intercept)	1.98 (1.68–2.33) 0.45 (0.34–0.60)		4.75 (4.30–5.24) 0.49 (0.38–0.64)		1.75 (1.11–2.76) 0.06 (0.04–0.11)		25.05 (22.67–27.67) 0.02 (0.01–0.04)		3.12 (2.64–3.69) 0.87 (0.65–1.16)	
Change 2012–2014	1.19 (0.98–1.45) 0.56 (0.38–0.82)	0.0744 0.0027*	0.99 (0.85–1.14) 0.46 (0.32–0.65)	0.8474 <0.0001*	1.03 (0.66–1.59) 0.94 (0.51–1.74)	0.9095 0.8542	1.43 (1.29–1.58) 4.81 (2.55–9.06)	**<0.0001** * **<0.0001** *	0.82 (0.67–1.01) 0.92 (0.65–1.29)	0.0650 0.6172

Zero-inflated regression models were used. Count part: modelling the amount of given blood products; Logit part: patient’s probability to receive blood products.

FFP: fresh frozen plasma; PCC: prothrombin complex concentrate; RBC: red blood cells.

a Zero-inflated Poisson regression model.

b Zero-inflated negative-binomial regression mode.

* Statistically significant.

In the ROTEM group, the usage of all the coagulation products was markedly higher than in the control group: the ROTEM group received platelets more often (33 vs 15 patients; *p* = 0.0007), FFP (23 vs 10 patients; *p* = 0.0147), fibrinogen (17 vs 5 patients; *p* = 0.00097) and PCC (22 vs 8 patients; *p* = 0.0039). Red blood cells were given as often (to as many patients) as in the control group (29 vs 25 patients; *p* = 0.247), but in smaller amounts at a time (3.5 vs 4.5 units/patient; *p* = 0.046) (Table 5).

**Table 5:** Blood product usage in the ROTEM group and in the control group

Count part Logit part	Platelets (units) ^a^		FFP (units) ^a^		Fibrinogen (units) ^a^		PCC (units) ^b^		RBC (units) ^a^	
	OR (CI)	*p*-Value	OR (CI)	*p*-Value	OR (CI)	*p*-Value	OR (CI)	*p*-Value	OR (CI)	*p*-Value
Control group (intercept)	3.20 (2.37–4.31) 0.39 (0.21–0.71)		5.80 (4.53–7.43) 0.25 (0.13–0.47)		2.11 (1.08–4.13) 0.11 (0.04–0.29)		45.0 (34.9–58.0) 0.17 (0.08–0.35)		4.45 (3.66–5.40) 0.77 (0.45–1.31)	
Change to ROTEM group	0.87 (0.60–1.26) 4.36 (1.86–10.2)	0.4670 0.0007*	0.83 (0.60–1.13) 2.88 (1.23–6.74)	0.2270 0.0147*	1.11 (0.52–2.36) 4.47 (1.44–13.9)	0.7879 0.0097*	0.95 (0.71–1.28) 3.88 (1.55–9.75)	0.7320 0.0039*	0.75 (0.56–0.99) 1.58 (0.73–3.41)	0.0455* 0.2470

Zero-inflated regression models were used. Count part: modelling the amount of given blood products; Logit part: patient’s probability to receive blood products.

FFP: fresh frozen plasma; PCC: prothrombin complex concentrate; RBC: red blood cells.

a Zero-inflated Poisson regression model.

b Zero-inflated negative-binomial regression model.

* Statistically significant.

## Discussion

The aim of this study was to retrospectively evaluate how implementing the ROTEM testing in our clinic in 2013 has influenced the usage of blood products and coagulation factors and, in addition, if it has had an effect on the re-operation rate, drainage volume or the length of stay in the ICU. We found that in the 2014 cohort compared to the 2012 cohort, there was a significant decrease in the postoperative blood drainage. The number of patients receiving platelets and FFP units also significantly decreased, but the use of PCC markedly increased from 2012 to 2014. When comparing the subgroup of the ROTEM tested patients with the matched control group, the ROTEM patients received coagulation products significantly more often.

In the 2014 cohort, the mean postoperative blood drainage volume was reduced in our patients compared to the 2012 cohort. The change did not, however, have an effect on the rate of postoperative re-sternotomies, which remained equal in both years. In the subgroup of the ROTEM tested patients, the reduction of drainage was only seen in a combination operation of CABG and valve. In the recent study of Kuiper et al (2019), the postoperative bleeding diminished distinctly when ROTEM was used. In our retrospective study, however, not all the patients were tested with ROTEM, as ROTEM was mostly used to assist transfusion decisions in the case of severe bleeding. Still, there is a clear change in blood transfusion practices before and after the introduction of the ROTEM. As ROTEM was used mostly in cases of severe bleeding or inadequate coagulation, the ROTEM patients received markedly more of the coagulation products than the control group. However, as the total use of coagulation products decreased in the 2014 cohort compared to the 2012 cohort, it is assumed that due to the ROTEM testing coagulation products were better targeted.

In the ROTEM group, a significantly shorter ICU length of stay was seen. However, this change was mostly due to the small number of patients in the control group who stayed in the ICU for several days (SD 116.6 hours). The median ICU stay was almost identical: 24.0 hours and 24.5 hours in the ROTEM group and controls, respectively. Although our results of the ICU stay are not very convincing, some studies have shown reduction in the ICU length of stay (Vasques et al 2017). However, such findings are quite impossible to obtain with such a small number of subjects as we had, and a cohort of not-excessively bleeding patients, most of them being stable enough to be discharged from the ICU on the first postoperative day.

The finding that fewer patients received platelets in 2014 cohort, despite a higher number of patients with ongoing clopidogrel, is noteworthy. As the ROTEM group received more platelets than the control group, it is also assumed that the usage of platelets was therefore better focused in the 2014 cohort. On the other hand, our ROTEM transfusion protocol readily suggests platelet use; if all the measures are within the limits, but there is still a problem with the coagulation, platelets are recommended. Similarly, our ROTEM protocol often suggests fibrinogen. As platelets and fibrinogen are frequently recommended in our ROTEM protocol, this may have some effect on their consumption. The increase in fibrinogen usage in ROTEM patients is a well-known phenomenon (Vasques et al 2017). The better control of bleeding is often seen, which is related to the increased use of fibrinogen (Vasques et al 2017).

In our unit, the treatment of perioperative bleeding in cardiac surgery patients is conducted by an anaesthetist and managed according to the general European principles (Kozek-Langenecker et al 2017). After 2013, ROTEM testing was used when there was a need for transfusions or problems with coagulation. Recent studies show increasing evidence that the use of ROTEM-based algorithms might reduce transfusions in cardiac surgery (Kozek-Langenecker et al 2017, Kuiper et al 2019, St-Onge et al 2018). In the study of Kuiper et al (2019), the use of FFP decreased and the use of tranexamic acid increased. In our hospital, however, 2500mg of tranexamic acid is routinely used for all the patients. As in the study of St-Onge et al (2018), we observed no change in the consumption of red blood cells. Despite this, the consumption of other blood products altered noticeably between the 2012 cohort and the 2014 cohort. Moreover, consistent with the above-mentioned studies, the FFP was administrated less often in 2014 (Kuiper et al 2019, St-Onge et al 2018). On the other hand, while the use of FFP was reduced, the use of PCC distinctly increased, which is reasonable, as they can be used in relatively similar situations and PCC is often more rapidly available (Nienaber et al 2011). In addition, the dilution can be a remarkable problem in large transfusions with FFP, and the usage of PCC then becomes more reasonable.

The ROTEM group received both FFP and PCC more often than the controls. The increased consumption of coagulation products in the ROTEM group is logical, for the ROTEM testing is often done in a situation where there is a problem with bleeding and coagulation. Since annual blood product utilisation appears to be decreasing, it can be assumed that the treatment was targeted to the right patients. Despite increased use of coagulation products in the ROTEM patients, the use of red blood cell units per patient had slightly decreased. This is an encouraging result, for the mortality risk has shown to be directly proportional to the number of transfused red blood cells units (Santos et al 2013).

We used cardiac surgery data retrospectively from two different six-month periods in 2012 and 2014 in Turku University Hospital where the ROTEM-guided transfusion algorithms were introduced in 2013. The groups were paired in terms of age, sex and operation type. However, there are some limitations related to retrospective data; some information was incomplete, and we had to exclude some patients due to missing values. Moreover, and unfortunately, ROTEM testing was only done thoroughly for a small part of the patients. The results might have been quite different if ROTEM had been done for all the patients in the 2014 cohort; the decrease in the blood product usage might have been even more visible. Based on clinical experience and patient documents, ROTEM was used more frequently for the patients with coagulation problems or in the case of severe bleeding. In the ROTEM group, there was significantly more clopidogrel users as well. Despite these weaknesses of the study, we managed to show that the blood product usage has changed after adopting ROTEM testing into practice. The total consumption of blood products reduced from the 2012 cohort to the 2014 cohort. Some decrease in drainage volumes was also seen. Our study suggests that ROTEM may have been beneficial in the treatment of bleeding patients. However, more research is needed to verify the results and to assess cost-effectiveness of ROTEM, and its impact on the patient care.

In conclusion, after the introduction of ROTEM, a reduction in total postoperative bleeding was seen. Also, the use of FFP and platelets decreased, whereas the use of PCC increased. In the subgroup of ROTEM tested patients, the usage of coagulation products was markedly higher than in the matched control group. In 2014, blood products were generally given more cautiously than in 2012, and the ROTEM assessments were often used to assist in the management of major bleeding. As the annual usage of coagulation products has decreased, it may be possible that ROTEM testing can help clinicians choose the most suitable coagulation products for patients who may bleed.

*No competing interests declared*
